# Effectiveness of a Single Prolonged Aerobic Exercise Session on Executive Function Task Performance in Physically Active Adults (21–70 Years of Age)

**DOI:** 10.3390/ijerph20042802

**Published:** 2023-02-04

**Authors:** Brandon A. Yates, Lawrence E. Armstrong, Elaine C. Lee, Frederick W. Unverzagt, Ekow Dadzie, Virgilio Lopez, Keith Williamson, Jakob L. Vingren, Ariela R. Orkaby

**Affiliations:** 1 Indiana Center for Musculoskeletal, Indiana University School of Medicine, Indianapolis, IN 46202, USA; 2 Indiana University Center for Aging Research, Regenstrief Institute, Inc., Indiana University School of Medicine, Indianapolis, IN 46202, USA; 3 Human Performance Laboratory, University of Connecticut, Storrs, CT 06269, USA; 4 Department of Psychiatry, Indiana University School of Medicine, Indianapolis, IN 46202, USA; 5 Vinson Health Center, Midwestern State University, Wichita Falls, TX 76308, USA; 6 Applied Physiology Laboratory, Department of Kinesiology, Health Promotion, and Recreation, University of North Texas, Denton, TX 76203, USA; 7 Department of Biological Sciences, University of North Texas, Denton, TX 76203, USA; 8 New England Geriatric Research, Education, and Clinical Center (GRECC), VA Boston Healthcare System, Boston, MA 02130, USA; 9 Massachusetts Veterans Epidemiology Research and Information Center (MAVERIC), VA Boston Healthcare System, Boston, MA 02130, USA; 10 Division of Aging, Brigham and Women’s Hospital, Harvard Medical School, Boston, MA 02115, USA

**Keywords:** cognition, physical activity, brain health, aging

## Abstract

We sought to examine the effectiveness of an acute prolonged exercise session on post-exercise executive function in physically active adults and to assess if age or pre-exercise cognitive performance was predictive of the magnitude of change in executive task performance. Self-registered cyclists were recruited prior to participating in a 161-km mass-participation cycling event. Cyclists were excluded if they had not previously participated in a similar endurance event, were young (<18 y), or were cognitively impaired (Mini Cog^TM^ < 3 units). Immediately after completing the exercise session, the time taken to complete Trail Making Test Part A and Part B (TMT A + B) was assessed. A faster time to complete the TMT A + B was observed after exercise (+8.5%; *p* = 0.0003; n = 62; age range = 21–70 y). The magnitude of change in TMT A + B performance (pre vs. post) was influenced by pre-exercise TMT A + B performance (r^2^ = 0.23, *p* < 0.0001), not age (r^2^ =0.002; *p* = 0.75). Prolonged exercise had a small-to-moderate effect on post-exercise compared to pre-exercise executive function task performance (*Cohen’s d* = 0.38–0.49). These results support the effectiveness of a single prolonged exercise bout to augment executive function in physically active adults, irrespective of age.

## 1. Introduction

The 2018 U.S. Health and Human Services Physical Activity Advisory Committee report present compelling evidence supporting the positive influence of chronic participation in moderate-to-vigorous physical activity or structured exercise on cognitive function across the human lifespan [1,2]. However, the novel recommendation from this report–promotion of single session or acute bouts of exercise to elicit cognitive benefits–was derived primarily from laboratory-based studies (i.e., behavior restricting), which limit the generalizability of those recommendations to real-world settings. Stated differently, while laboratory-based studies provide evidence of the *efficacy* of a single aerobic exercise session, the ability to deduce the potential *effectiveness* (i.e., the effect observed in real-world settings) is limited under these conditions. Indeed, under real-world conditions, adults likely incorporate personalized behavioral strategies to reduce the onset of perceptual or muscular fatigue (e.g., listening to music, *ad libitum* fluid consumption, rest breaks, social interaction, etc.) [3] and increase enjoyment, which conceivably results in increased compliance and adherence to evidence-based exercise guidelines [4]. Thus, with the growing recognition of exercise as medicine [5], it is imperative that, similar to pharmacological prescriptions, consideration be given as to how exercise/physical activity prescriptions translate from clinical settings to real-world environments and the various factors that influence the effectiveness of single sessions.

The c extant literature suggests that several factors may influence the cognitive-enhancing benefit of a single exercise/physical activity bout: age, duration, and pre-exercise cognitive task performance [6,7,8,9,10,11,12,13,14]. Chief among these is age, in that older adults tend to achieve greater post-exercise cognitive improvements than younger adults; however, few studies have examined different age groups under similar conditions [6,7,8,9,10,11,12,13]. This has led to a paucity of data on middle-aged adults. Further, a minimal exposure/dosage of 10 min has been recommended to observe an exercise-induced cognitive enhancement [6,7,8,9,10,11,12,13], but despite data showing that longer duration (i.e., 45 min) may be superior to shorter durations [15], data on prolonged duration exercise (e.g., marathon or ultra-endurance events) is lacking. As such, uncertainty remains about whether "mega-doses" of exercise also promote post-exercise cognitive enhancement. Further, a recent systematic review and meta-analysis of individual participant data by Ishihara and colleagues [14] showed that post-exercise effects on executive function were largely driven by pre-exercise task performance. Thus, a plausible confounder in prior studies examining the effect of an acute exercise bout on cognition may be heterogeneity in baseline task performance. Hence, accounting for this variable is encouraged in future research.

To our knowledge, only two studies have investigated the influence of a single prolonged exercise bout (>2 h) on cognitive function [16,17]. However, these 2 studies produced equivocal results, potentially due to small sample sizes and differences in the study population. Therefore, the primary objective of the present study was to examine the effectiveness of a single prolonged aerobic exercise session on post-exercise executive function task performance in physically active adults and to examine the association of age and pre-exercise executive function task performance with the magnitude of change in executive function task performance (pre vs. post). We hypothesized that a single prolonged aerobic exercise session would improve post-exercise executive function task performance in a cohort of physically active adults and that both pre-exercise cognitive task performance and age would be associated with the magnitude of post-exercise executive function task improvement.

## 2. Material and Methods

### 2.1. Study Population

This prospective and pragmatic cohort study was reviewed and approved by the Institutional Review Board for Human Studies at an academic university in Texas (USA). Participants were recruited via verbal solicitation within the exhibit hall 24–48 h prior to a mass participation cycling event. All participants provided written informed consent before completing a medical history questionnaire, which the study team assessed for the following exclusionary criteria: <18 or >90 years of age, cognitively impaired as determined by the Mini Cog^TM^ assessment (excluded if the score was <3/5, 1 point each for 3-word recall and 2 points for correct clock draw), current musculoskeletal injury, fluid balance altering illness or medication (e.g., diuretic), dietary manipulation that omitted one nutrient or class of nutrients (e.g., vegetarian or vegan), current smoker or tobacco user, or had not previously completed a 161 km cycling event.

At enrollment, participants’ anthropometric data were collected (body weight, height, and body fat percentage via a three-site measurement with calibrated skinfold calibers). Participants’ habitual gait speeds over a 4 m distance were measured as a surrogate marker of cardiorespiratory fitness, given its strong relationship with peak oxygen consumption in aging adults (r = 0.74) [18]. The fastest of two trials was used for statistical analysis. Lastly, participants were familiarized with the Borgs Ratings of Perceived Exertion scale (RPE; scores ranged from 6 representing no exertion at all to 20 representing maximal exertion), which correlates strongly with physiological exercise intensity parameters [19].

### 2.2. Exercise Duration and Environmental Conditions

All participants pre-registered for and completed the outdoor mass participation 161 km cycling event in Wichita Falls, Texas, USA, in August of 2017 using their own bicycles. The weather conditions on the day of the event were: Temperature = 21–31 °C (range); Dew point = 20–22 °C (range); Peak wind speed = 22.5 km/h (mostly <16 km/h throughout the event); and Precipitation = 0. On the event day, cyclists began to assemble as early as 4 AM, and the event officially started at 7 AM. In the event, a total of 3373 (men = 2821; women 552) cyclists completed the mass participation event at an average time of approximately 7 h.

### 2.3. Cognitive Assessments

The Trail Making Task (TMT) was selected because it can be administered easily outside of laboratory settings, is valid and reliable [20], and measures executive functions that are sensitive to acute aerobic exercise bouts [14,20]. To attenuate the influence of a learning effect, participants were familiarized with the TMT battery 24–48 h prior to the event. Familiarization consisted of each participant performing the TMT battery one time under the instruction of a study team member. The TMT battery consists of a Part A (TMT-A) and Part B (TMT-B). The TMT-A is a simple cognitive task that requires the participant to connect scattered encircled numbers (1–25) in order and as quickly as possible on an 8 by 10-inch sheet of paper and measures psychomotor speed. The TMT-B is a more complex task that requires the participant to connect an encircled number to an encircled letter to a number and to a letter, alternately and in order, as quickly as possible (i.e., 1 to A, 2 to B, etc.), and measures cognitive flexibility. Participants were provided a seat, clipboard, and table in a well-lit area to complete the TMT battery. The total time to complete TMT-A and -B was recorded with a handheld stopwatch. On the event day, participants completed the TMT battery in sequential order (A before B) before and after completion of the cycling event (within 5–15 min).

### 2.4. Outcome Variables

The primary outcome was the total time it took to complete the TMT battery and individual parts (i.e., A and B) before and following a single prolonged exercise session.

### 2.5. Statistical Analysis

Pre-exercise cognitive outcome variables were screened, and values ≥3 standard deviations from the mean were reviewed for veracity and precision of measurement compared to the familiarization trial. The Shapiro-Wilk test for normality and Levene’s test for homogeneity were used. Changes in the time to complete the TMT battery (pre vs. post) were evaluated with a paired sample *t*-test for which *Cohen’s d* effect sizes were derived. A linear regression analysis was used to assess if age or pre-exercise executive function task performance was associated with the magnitude of change in executive function task performance (pre vs. post). Statistical significance was set a *p* < 0.05. Data presented are (mean difference [95% Confidence Interval (CI)]) unless designated otherwise. All analyses were performed using SPSS Statistics, Version 27.0 (IBM Corp. 2020, Armonk, NY, USA).

*Power analysis*. To detect a statistically significant effect of prolonged exercise on time to complete TMT A + B at an α level set of *p* < 0.05, power of 0.80, an effect size of 0.4, and using a paired sample student’s *t*-test, a sample size of 52 participants was required (G*Power, V.3.1.9, Aichach, Germany). To account for a possible 20% attrition, we allowed up to 63 participants to be enrolled in this study.

## 3. Results

*Participant characteristics and exercise performance*. A total of 63 participants were enrolled in this study. One participant was removed prior to statistical analyses due to an extreme pre-exercise value significantly slower than during familiarization (TMT A + B = 128 vs. 233 sec; familiarization vs. pre-exercise for the excluded participant). Therefore, data is provided on 62 participants unless stated otherwise. The majority of the sample had robust physical function, as determined by their habitual gait speed (1.07 ± 0.16 m⋅s^−1^ (mean ± standard deviation)), were >50 years old (66.1%; range 21–70 years of age), and identified as male (97%, Table 1). At the completion of the event, the average finish time was 373 ± 80 min (n = 53; 11 participants were lost to follow-up) with a final reported RPE of 16 ± 2 arbitrary units (n = 60; 2 participants were lost to follow-up).

**Figure 1:**
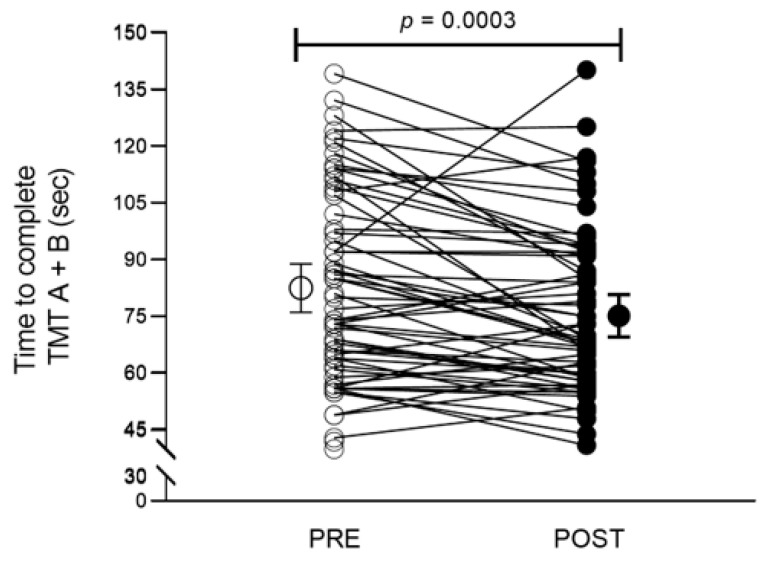
Participants completed the Trail Making Test A + B faster after an acute prolonged exercise session (n = 62; 21–70 years of age). Data analyzed via paired sample *t*-test.

**Figure 2:**
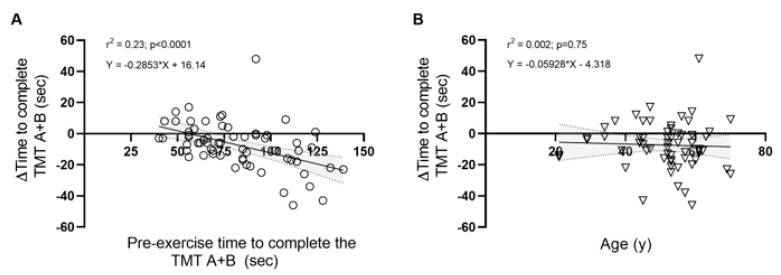
Baseline Trail Making Test A + B performance (n = 62) accounted for 23% of the variance in the magnitude of change following the prolonged exercise bout. Linear regression analysis of the association between the change in time to complete the TMT A + B (post vs. pre) (∆TMT A + B; independent variable) with (**A**) pre-exercise time to complete the Trail Making Test Part A + B and (**B**) age.

*Cognitive Outcomes*. We observed a significant improvement (i.e., decrease in time) in the time to complete the TMT A + B compared to pre-exercise performance (−7 sec [95% CI: −11 to −4]; *p* = 0.0003; Figure 1). Small to moderate effect sizes were observed in time to complete TMT A, TMT B, and TMT A + B (*Cohen’s d* = 0.38–0.49; Table 2). Regression analysis revealed that pre-exercise performance in the TMT A + B explained 23% of the variance in the magnitude of change (post vs. pre) in executive function task performance after the event (r^2^ = 0.23, F_1,60_ = 17.92, *p* < 0.0001; Figure 2), but not age (r^2^ = 0.002, F_1,60_ = 0.11, *p* = 0.75; Figure 2).

## 4. Discussion

The primary novel findings of this study are threefold. First, after a single prolonged exercise session, lasting roughly 6 h, participants performed executive function tasks significantly faster compared to pre-exercise. Second, contrary to our hypothesis, the magnitude (i.e., mean change in time to complete tasks) of cognitive enhancement was not moderated by age but rather by pre-exercise executive function task performance. Third, we observed a positive small-to-moderate effect size for a single prolonged exercise session on improving immediate post-exercise executive function in an ecologically relevant setting comparable to the effect sizes reported in data derived from laboratory-based studies [9,10,12,21]. Collectively, these findings demonstrate a positive impact of a single session of a prolonged exercise session on executive function.

To our knowledge, only two prior studies have examined the influence of a prolonged aerobic exercise session on post-exercise executive function task performance. In a similar pragmatic study, Benefer et al. used the TMT battery to examine changes (pre vs. post) in cognition following a prolonged exercise (31.6 ± 9.7 km) in 33 physically active walkers and runners (mean age = 43 years) [16]. Interestingly, the exercise duration (~6 h) and post-exercise TMT-A and -B completion times were comparable to our study, 2 (−9%) and 4 (−10%) seconds for the TMT-A and -B, respectively [16]. Conversely, in a laboratory setting, Grego et al. examined the influence of 3 h of moderate-intensity cycling on a complex executive function task in a smaller sample of young endurance-trained adults and recreational exercisers (mean age = 30 years; n = 8) [17]. The authors concluded that exercise-induced cognitive enhancing benefits were achieved after 1 and 2 h of exercise with diminishing results following the last hour of exercise. However, the lack of an exercise-induced cognitive enhancing benefit beyond 2 h is likely attributable to the small sample size and possibly heterogeneity in baseline cognitive task performance [17].

A major strength of this investigation is our moderate sample size compared to prior studies. Further, participants were not forced or counseled to perform a prescribed exercise regimen (i.e., they were self-registered for the event and were free to complete the bout at a self-selected pace/intensity) and utilized their own exercise behavioral strategies (e.g., listening to music, individualized diet and hydration regimens, social engagement, and/or ad libitum rest breaks) which increases the external validity of our results. However, this approach subsequently reduced our internal validity, and therefore caution must be taken in extrapolating our findings to other populations. For instance, using a pragmatic design limited our ability to feasibly account for factors that likely mitigate fatigue during the exercise bout and improve cognition: nutritional supplementation, fluid intake, ergogenic aids, and sleep hygiene [22,23,24,25]. Additionally, most of our participants identified as male; therefore, precautions are needed in generalizing our findings to women [26]. In this regard, gender as a moderator of the exercise-cognition relationship remains equivocal, with data supporting both reduced and augmented post-exercise cognitive enhancement [10]. Lastly, there was no control group (i.e., a group performing the cognitive battery but not exercising); therefore, we do not know the extent to which practice effects linked to simple exposure to the cognitive tests on 3 occasions (fam, pre-, and post-exercise), separate from exercise-induced aerobic effects, produced the after-exercise changes in TMT completion time.

In conclusion, the key findings from this ecological investigation provide novel evidence showing that healthy, physically active adults (21–70 y) can achieve exercise-induced cognitive enhancement after a single prolonged aerobic exercise bout, regardless of age. This finding extends the understanding of the therapeutic benefits of aerobic exercise on cognitive function during adulthood [1,2,6]. These results encourage future research to identify what behavioral strategies (before or during exercise) influence the magnitude of cognitive enhancement, how those strategies influence exercise duration, and the biological mechanisms underpinning augmented executive function task performance following a single exercise session.

## Figures and Tables

**Table 1:** Participant demographics, pre-exercise cognitive function, and exercise performance.

Demographics	Entire Sample (n = 62)
*Characteristic*	
Age, y	51 (11)
20–29, #	4 [6.5]
30–39, #	4 [6.5]
40–49, #	13 [20.9]
50–59, #	31 [50.0]
60–69, #	8 [12.9]
70, #	2 [3.2]
Female gender, #	3 [4.8]
Weight, kg	87.2 (12.4)
Height, cm	177 (6)
BMI, kg/m^2^	27.9 (3.4)
Body Fat, %	13.8 (5.3)
Habitual gait speed, m/s	1.07 (0.16)
*Cognitive Performance*	
TMT-A, sec	27 (10)
TMT-B, sec	56 (18)
*Exercise performance*	
Total exercise time, min ^a^	373 (80)
RPE, *arbitrary units* ^b^	16 (2)

Values are mean (standard deviation) or number [percentage]; ^a^ denotes data provided on n = 53, and ^b^ denotes n = 60 due to loss to follow-up; # = total number of participants in the group out of 62. Abbreviations: Trail Making Test Part A (TMT-A) and Part B (TMT-B), Body Mass Index (BMI), and Rating of Perceived Exertion (RPE).

**Table 2:** Effect sizes for comparisons of time to complete cognitive task (pre vs. post-exercise).

Cognitive Tack	*Cohen’s d* Effect Size
Trail Making Test Part A, sec	0.38 [0.12, 0.63]
Trail Making Test Part B, sec	0.34 [0.01, 0.60]
Trail Making Test Part A + B, sec	0.49 [0.22, 0.75]

All values are effect size [95% Confidence Interval].

## Data Availability

Data will be made available upon request via email to corresponding author.
